# Long-term effectiveness and safety of infliximab, golimumab and golimumab-IV in rheumatoid arthritis patients from a Canadian prospective observational registry

**DOI:** 10.1186/s41927-020-00145-4

**Published:** 2020-09-19

**Authors:** Proton Rahman, Philip Baer, Ed Keystone, Denis Choquette, Carter Thorne, Boulos Haraoui, Andrew Chow, Rafat Faraawi, Wojciech Olszynski, John Kelsall, Emmanouil Rampakakis, Allen J. Lehman, Francois Nantel

**Affiliations:** 1 Memorial University, St. John’s, NL Canada; 2 Scarborough, Canada; 3 University of Toronto, Toronto, ON Canada; 4 Institut de Rhumatologie de Montréal, Montreal, QC Canada; 5 Newmarket, Canada; 6 Mississauga, ON Canada; 7 McMaster University, Hamilton, ON Canada; 8 University of Saskatchewan, Saskatoon, SK Canada; 9 Providence Health, Vancouver, BC Canada; 10 JSS Medical Research, Montreal, QC Canada; 11 Janssen Inc., 19 Green Belt Dr., Toronto, ON M3C 1N9 Canada

**Keywords:** Rheumatoid arthritis, Registry, Infliximab, Golimumab, Effectiveness, Safety

## Abstract

**Background:**

Long-term clinical registries are essential tools to evaluate new therapies in a patient population that differs from those in randomized clinical trials. The objectives are to describe the profile of rheumatoid arthritis (RA) patients treated with anti-TNF agents in Canadian routine care.

**Methods:**

RA patients eligible for treatment with Infliximab (IFX), golimumab (GLM) or intravenous golimumab (GLM-IV) as per their respective Canadian product monographs were enrolled into the BioTRAC registry between 2002 and 2017. Study visits occurred at baseline and every 6 months thereafter. Effectiveness was assessed by changes in disease activity. Safety was evaluated by the incidence of adverse events (AEs) and drug survival.

**Results:**

Of the 890 IFX-, 530 GLM- and 157 GLM-IV-treated patients, the proportion of females ranged from 77.0–86.6%, the mean ages from 55.8–57.7 and the mean disease duration from 6.5–8.6 years. A significant decrease in baseline disease duration and disease activity parameters (DAS, TJC, SJC, HAQ, AM stiffness, MDGA, PtGA, CRP, ESR) was observed over time. Treatment with IFX, GLM- and GLM-IV significantly improved all disease parameters over time. The incidence of AEs was 105, 113 and 82.6 /100 PYs and the incidence of SAEs was 11.7, 11.2 and 4.68 /100 PYs for IFX, GLM- and GLM-IV-treated patients, respectively.

**Conclusion:**

Differences in baseline characteristics between patients treated with an anti-TNFs over time shows the evolution of treatment modalities over time. All treatments significantly reduced disease activity and improved functionality in a similar fashion. The incidence of adverse events was consistent with the safety profiles of IFX and GLM.

**Trial registration:**

ClinicalTrials.gov Identifier: NCT00741793 (Retrospectively registered on August 26, 2008).

## Background

Rheumatoid arthritis (RA) is a chronic, systemic inflammatory disease characterized by a symmetric, progressive inflammatory synovitis of the joints, leading to radiographic erosion, pain, functional disability, reduced quality of life and increased mortality [1]. Based on National and International treatment guidelines [2, 3], short-term glucocorticoids are recommended alongside disease-modifying antirheumatic drugs (DMARDs), specifically methotrexate (MTX), while biologic DMARDs (bDMARDs) are recommended after 3 months of failed treatment with at least 2 conventional DMARDs [2, 3]. Since the approval of the first bDMARDs, the anti-TNF agents infliximab (IFX) and etanercept, several new agents and strategies have been introduced for the treatment of moderate to severe RA [3].

These guidelines predominantly use data from randomized clinical trials (RCTs) which, although designed to minimize potential biases, are carried out in selected populations which usually differ from patients treated in a real-world setting [4]. RCTs typically involve a small number of patients and represent only a limited spectrum of the patients seen in real-life clinical practice. In addition, the time of exposure to the drugs and controls is usually limited. Therefore, RCTs cannot answer important questions concerning long term safety or therapeutic strategy, and data from RCTs cannot easily be extrapolated to daily practice [5]. Despite their methodological limitations, observational studies allow the investigation of the long-term effectiveness and safety of new therapies and/or treatment strategies in a larger, more representative populations.

Here, we report long-term data on the profile of RA patients treated with several anti-TNF bDMARDs in Canadian routine clinical care over time, as well as describe their real-world effectiveness and safety over a 16-calendar year period.

## Methods

### Study design

The Biologic Treatment Registry Across Canada (BioTRAC; NCT00741793) was a prospective, multi-center, industry-funded study that collected real-world clinical, laboratory, safety, and patient-reported data among ankylosing spondylitis, psoriatic arthritis, and RA patients treated with IFX, golimumab (GLM), intravenous golimumab (GLM-IV) or ustekinumab during routine care in academic and community centers in Canada between 2002 and 2018. BioTRAC was originally designed and launched in February 2002 as an effectiveness and safety registry for RA patients treated with IFX. Patients or the public were not involved in the design, or conduct, or reporting, or dissemination of this study. The registry was amended in 2005 to include IFX-treated patients with ankylosing spondylitis, and further expanded in 2006 to psoriatic arthritis. In 2010, patients treated with GLM were included. Finally, the registry was amended once more in 2014 to include RA patients treated with GLM-IV and psoriatic arthritis patients treated with UST. Additional details on the study design and an interim analysis of the IFX RA cohort have been previously published [6]. Prior to enrollment, patients were required to provide written informed consent to participate. Ethics approval was obtained from a central Research Ethics Board (IRB Service, Ontario, Canada) for private practices, and from respective Research Ethics Boards for institutional sites. The study was conducted in accordance with the Declaration of Helsinki and adheres to CONSORT guidelines. Data from this study were presented at the Canadian Rheumatology Association [7], PANLAR [8] and EULAR [9] 2019 conferences.

### Patient population

Rheumatology patients, either bio-naive (2002–2006) or with ≤1 prior biologic agent exposure (2006–2018), were enrolled and followed for up to 14 years with a study visit at baseline and every 6 months thereafter (a 2-month visit was also included from 2002 to 2006). From 2006 to 2009, additional inclusion criteria included SJC > 10 or CRP > 0.8 mg/dL or ESR > 30 mm/hr.

Patients treated with IFX were enrolled until May 2015 when the pre-specified recruitment number of 1500/drug across diseases was met and were followed until Jan 2017. Enrolment for GLM- and GLM-IV-treated patients was stopped in Jun 2017 when the overall recruitment number of 3000 was met, and they were followed until Jun 2018. For the purposes of this analysis, patients with RA who initiated IFX, GLM or GLM-IV treatment were included. All analyses were conducted in the full analysis set comprising patients receiving treatment without major eligibility violations.

### Data collection

The following clinical, laboratory and patient-reported outcomes (PROs) were collected as per routine care at baseline and every 6 months thereafter: tender joint count based on 28 joints (TJC28), swollen joint count based on 28 joints (SJC28), Disease Activity Score 28 (DAS28), Health Assessment Questionnaire Disease Index (HAQ-DI), patient (PtGA) and physician (MDGA) global assessment of disease activity, C-reactive protein (CRP), erythrocyte sedimentation rate (ESR), morning (AM) stiffness, and pain. Target-specific outcomes, specifically SDAI remission (≤3.3) and low disease activity (LDA; ≤11) were calculated from raw scores. Safety was assessed with the incidence of treatment-emergent adverse events (AEs). As of 2014, due to changes in regulatory requirements, discontinuation due to unusual failure of efficacy (attributed to the product itself) started being reported as an AE of special interest.

### Statistical analysis

The current study includes data from two distinct statistical analysis plans. The first plan covered the IFX cohort and was filed in May 2018. The second plan covered the remainder of cohort and included patients treated with either GLM or GLM-IV. Since the investigators had already been exposed to the IFX data, a decision was made not to do any statistical analysis comparing the IFX cohort to the other patients. Nonetheless, comparative data is presented therein as it provides an interesting vision of how patients evolved over the years and how each drug was used. To that effect, a stratified analysis of patient baseline profiles was conducted based on enrolment period, specifically 2002–2004, 2005–2008, 2009–2012, 2013–2015 and 2016–2017.

All outcomes were assessed descriptively using the median and/or mean and standard deviation (SD), 95% confidence intervals (CI) of the mean for continuous variables, and frequency distributions for categorical variables. Variations in patient demographics and baseline characteristics across enrolment periods were assessed using the Wilcoxon Mann Whitney test for continuous variables and the Chi-square or Fisher’s exact test for categorical variables.

Kaplan-Meier survival analysis was used to assess time to discontinuation. AEs were coded using the Medical Dictionary for Regulatory Activities (MedDRA version 20.0), and the proportion of patients who experienced an AE along with incidence rates were summarized by preferred term (PT). Statistical analyses were conducted with SPSS 24.0 (SPSS Inc., Chicago, IL) and SAS 9.4 (SAS Institute, Cary, NC, USA).

## Results

Patient demographics and baseline characteristics are presented in Table 1. Of the 890 IFX-, 530 GLM- and 157 GLM-IV-treated patients, the proportion of females ranged from 77.0–86.6%, the mean age from 55.8–57.7 years and the mean disease duration from 6.5–9.8 years. Most patients were bio-naive. Patients treated with IFX received a mean (SD) dose of 3.4 (0.57) mg/Kg, over a median (min-max) of 13 (1–114) infusions representing a total exposure of 2714 patient years (pt.yrs) (mean patient follow-up: 3 years). All GLM-treated patients started at the 50 mg dose and received a median (min-max) of 16 (1–92) injections representing a total exposure of 1077 pt.yrs. (mean patient follow-up: 2 years). One patient received at least one 100 mg dose, 11 patients (2.1%) received 50 mg injections at shorter than q28 days intervals while 82 patients (15.6%) received 50 mg injections at q28–32 days intervals throughout study. For GLM-IV, the mean (SD) dose was 1.97 (0.56) mg/Kg over a median (min-max) of 11 (1–29) infusions representing a total exposure of 257 pt.yrs. (mean patient follow-up: 1.6 years).

**Table 1:** Patient demographics and baseline characteristics

	IFX	GLM	GLM-IV
**Number of Patients**	**890**	**530**	**157**
**Female Gender, n (%)**	773 (86.8%)	404 (76.2%)	121 (77.0%)
**Mean (SD) Age, years**	55.8 (13.5)	57.7 (13.0)	56.3 (12.3)
**Mean (SD) Weight, Kg**	75.4 (19.22)	76.8 (19.4)	78.4 (21.8)
**Positive Rheumatoid Factor, %**	68.4%	60.4%	58.6%
**Disease duration, years**			
Mean (SD)	9.8 (9.98)	8.0 (7.61)	6.5 (8.76)
Median	6.0	4.9	6.0
**Number of previous DMARDs**			
Mean (SD)	2.1 (1.41)	2.3 (1.08)	2.5 (0.97)
**Previous Therapies, %**			
DMARDs	87.2%	94.5%	98.7%
NSAIDs	59.7%	48.3%	54.8%
Corticosteroids	47.9%	52.8%	46.5%
Methotrexate	70.4%	84.7%	92.4%
**Concomitant Therapies, %**			
DMARDs	89.3%	88.6%	88.5%
NSAIDs	53.4%	43.8%	49.0%
Corticosteroids	36.9%	33.0%	28.7%
Methotrexate	71.1%	67.4%	68.2%
**Bio-naive, %**	93.7%	86.2%	80.3%
**DAS 28 CRP**^**a**^	5.3 (1.37)	4.5 (1.2)	4.1 (1.0)
**DAS 28 ESR**^**a**^	5.7 (1.49)	4.7 (1.40)	4.4 (1.16)
**TJC**^**a**^	12.3 (8.11)	9.5 (7.0)	9.2 (6.6)
**SJC**^**a**^	10.4 (7.04)	8.1 (5.7)	6.7 (4.8)
**PtGA**^**a**^	60.2 (24.12)	56.8 (25.2)	59.2 (25.2)
**MDGA**^**a**^	6.4 (2.15)	5.9 (2.2)	5.2 (2.5)
**HAQ**^**a**^	1.6 (0.70)	1.3 (0.7)	1.3 (0.7)
**Pain, VAS**^**a**^	57.2 (23.99)	55.2 (25.6)	58.2 (28.0)
**CRP, mg/L**^**a**^	18.2 (23.42)	15.4 (31.4)	20.1 (37.4)
**ESR, mm/hr**^**a**^	32.2 (24.16)	24.2 (20.6)	26.4 (18.6)
**Morning stiffness, min**^**a**^	65.3 (45.51)	54.4 (43.8)	60.3 (45.7)

^a^Mean (SD)

**Fig. 1:**
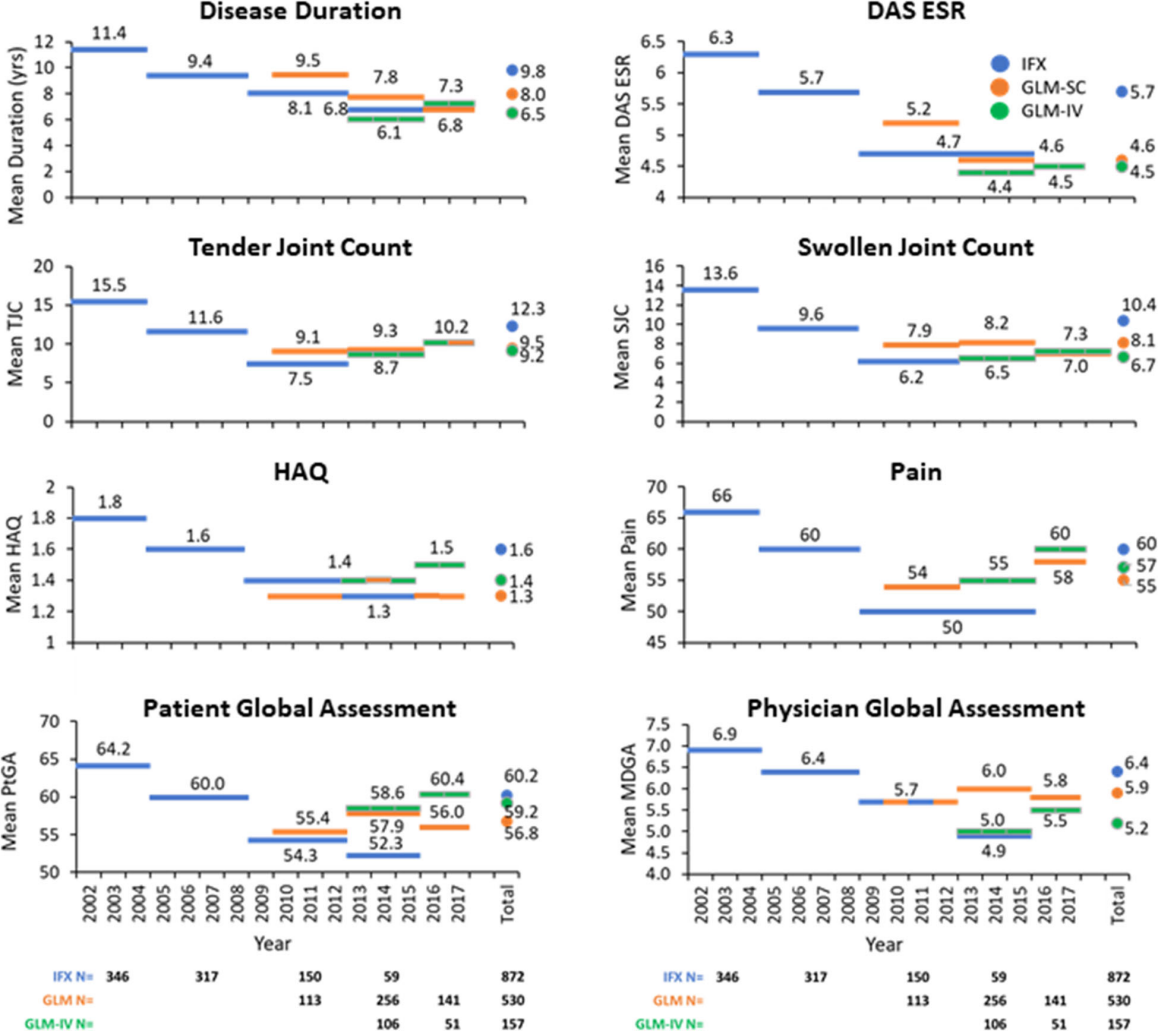
Evolution of baseline characteristics over time

As shown in Fig. 1, a significant decrease in baseline disease duration was observed in IFX-treated patients over the index year (*p* < 0.001). A similar reduction was also observed in baseline disease activity scores (DAS28 ESR, TJC, SJC, HAQ, AM stiffness, MDGA, PtGA, CRP, ESR) over the index year (Fig. 1 and Supplementary Material). In contrast, baseline disease duration and activity scores in the GLM- and GLM-IV-treated patients remained stable between 2010 and 2017. Interestingly, baseline disease duration and some of the disease activity scores (DAS28 ESR, TJC, SJC, PtGA, Pain, CRP, ESR) were higher in GLM-treated patients from the 2010–2012 time period when the drug was first introduced compared in IFX-treated patients despite the mean MDGA and HAQ being the same (Fig. 1).

**Fig. 2:**
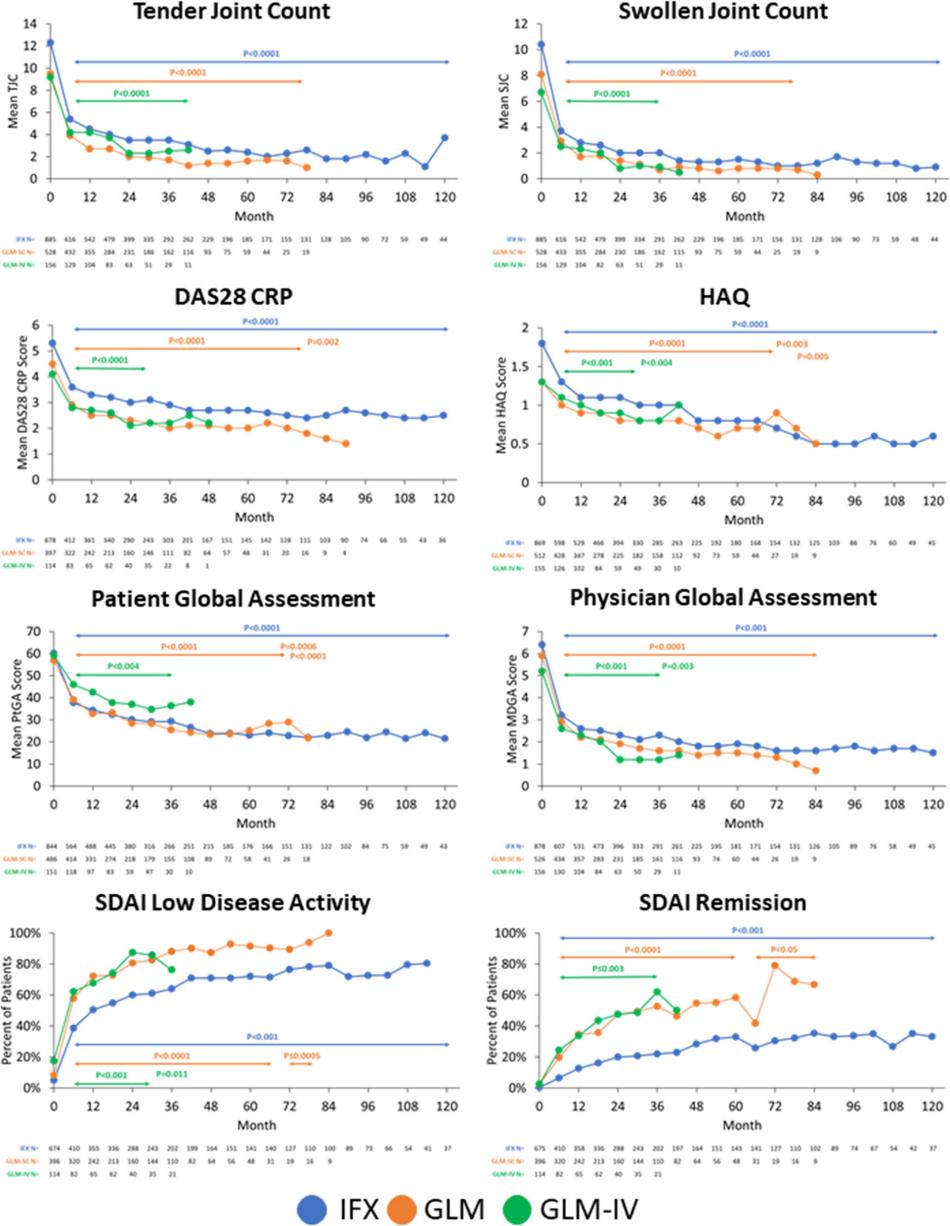
Effect of treatment with IFX, GLM and GLM-IV on disease parameters over time. Observed data with X-axis cut at 120 months for clarity (goes up to 168 months for IFX; *n* = 4). P value vs baseline

**Fig. 3:**
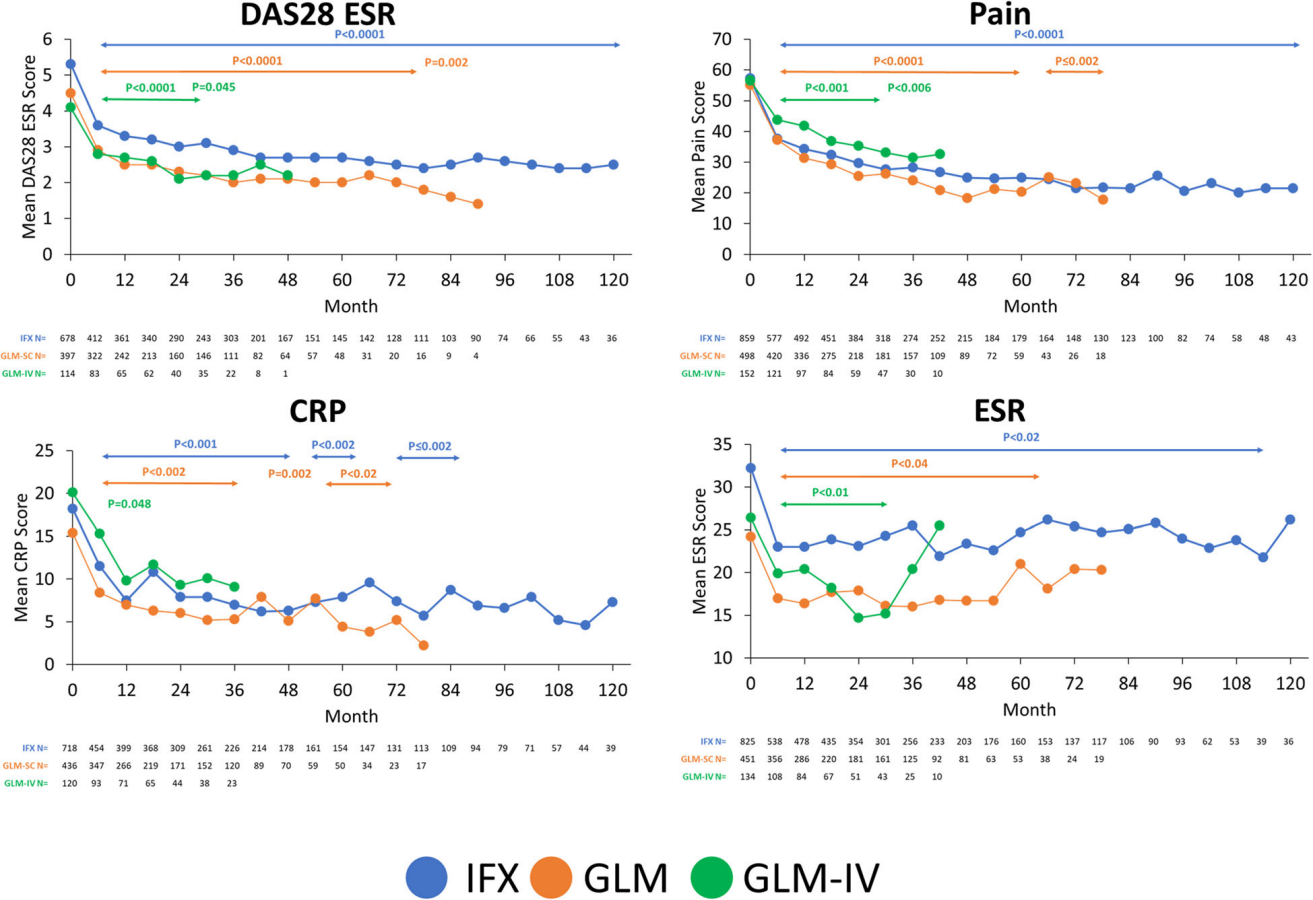
Effect of treatment with IFX, GLM and GLM-IV on disease parameters over time. Observed data with X-axis cut at 120 months for clarity (goes up to 168 months for IFX; *n* = 4). *P* value vs baseline

Treatment with all three anti-TNFs significantly improved TJC, SJC, DAS28 CRP, HAQ, PtGA and MDGA scores from baseline to 6 months and up to 120, 78 and 42 months for IFX, GLM and GLM-IV, respectively (Fig. 2). A similar effect was also observed for DAS28 ESR, pain, CRP and ESR (Fig. 3). However, achievement of target-specific outcomes appeared to differ between agents. Indeed, the proportion of patients in SDAI remission at 12, 24 and 36 months reached 16.2, 20.8 and 22.8% in IFX-patients; 34.7, 47.5 and 52.7% in GLM-patients and 33.8, 47.5 and 61.9% in GLM-IV-patients (Fig. 2). Similar patterns were observed with DAS28 remission and with CDAI LDA and remission (not shown).

**Fig. 4:**
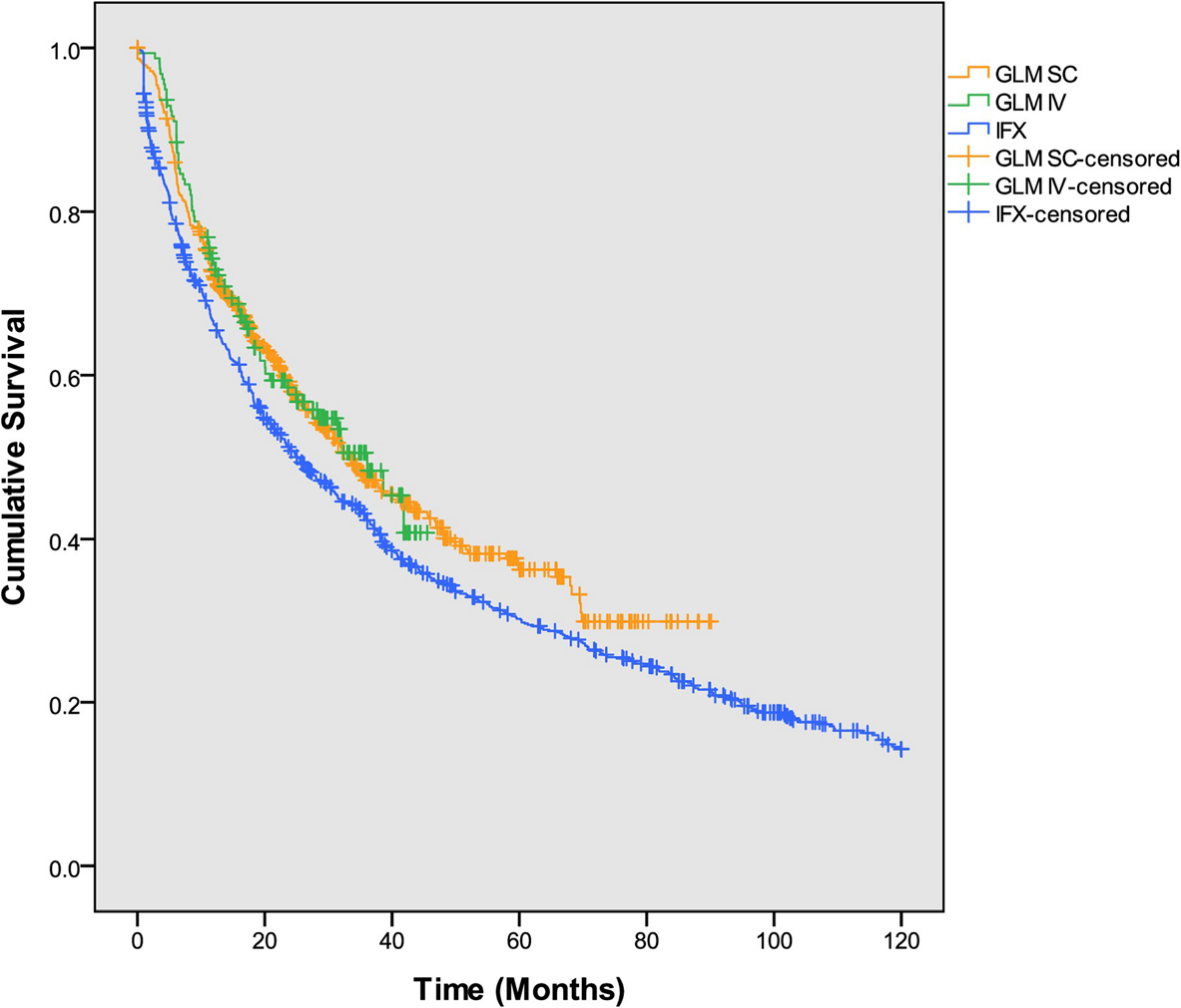
Kaplan-Meier drug survival analysis

The proportion of patients who discontinued treatment were 74.0% over a mean 3.0 years of exposure to IFX, 65.6% over 2.0 years of exposure to GLM and 45.2% over 1.6 year of exposure to GLM-IV. The median time to discontinuation was 24.9, 33.4 and 36.1 months for IFX, GLM and GLM-IV, respectively (Fig. 4). The reasons for discontinuations are shown in Table 2.

**Table 2:** Discontinuations and reasons for discontinuations*

	IFX	GLM	GLM-IV
**Total discontinuations (n/N, %)**	659/890, 74.0%	280/530, 65.6%	71/157, 45.2%
**Exposure (Total, Mean pt. yrs)**	2714, 3.0	1077, 2.0	257, 1.6
**Reason for discontinuation (n, %**^**a**^**)**			
Patient withdrew consent	58, 8.8%	25, 8.9%	4, 5.6%
Adverse event	116, 17.6%	33, 11.8%	10, 14.1%
Lost to follow-up	25, 3.8%	28, 10.0%	7, 9.9%
Financial reasons	14, 2.1%	4, 1.4%	0, 0.0%
Complete response	10, 1.5%	4, 1.4%	0, 0.0%
Disease progression	75, 11.4%	14, 5.0%	2, 2.8%
Lack of response	45, 6.8%	67, 23.9%	22, 31.0%
Loss of response	65, 9.9%	46, 16.4%	6, 8.5%
Unusual lack of efficacy	0, 0%	1, 0.4%	0, 0%
Geographic issues	24, 3.6%	3, 1.1%	2, 2.8%
Patient switched to another therapy	32, 4.9%	15, 5.4%	9, 12.7%
Did not meet entry criteria	1, 0.2%	0, 0.0%	0, 0.0%
Other	191, 29.0%	40, 14.3%	8, 11.3%
Missing	3, 0.5%	0, 0%	1, 1.4%

^a^Proportions based on number of discontinued patients

AEs were reported for 61.5, 67.4 and 59.2% (105, 113 and 82.6 events/100 PYs) and SAEs for 21.2, 15.5 and 3.8% (11.7, 11.2 and 4.68 events/100 PYs) covering 2714, 1077 and 257 years of exposure for IFX, GLM and GLM-IV-treated patients, respectively (Tables 3, 4 and 5). The most frequently occurring AEs were arthralgia and upper respiratory tract infection (> 5%). The most common serious infection was pneumonia. Sixty (6.7%) IFX-treated patients discontinued IFX due to an SAE. For GLM- and GLM-IV-treated patients, discontinuation due to an SAE occurred in 20 (3.8%) and 2 (1.3%) patients, respectively. There were 8 cases of opportunistic infections (including a new onset disseminated TB) in IFX-treated patients while none were observed in any GLM- or GLM-IV-treated patients. The incidence of malignancies, serious and opportunistic infections are further described in Table 6. In summary, the incidence rate of malignancies was similar between IFX- and GLM-treated patients (1.87/ and 2.41/100 pt.yrs., respectively) while only one case was reported in GLM-IV patients. There were three pregnancies in IFX-treated patients and two in GLM-treated patients (with 1 induced labor and 1 post-partum hemorrhage).

**Table 3:** Adverse events occurring in ≥4% of patients per agent

	IFX (***N*** = 890)				GLM (***N*** = 530)				GLM-IV (***N*** = 157)			
Exposure (Total, Mean pt.yrs)	2714, 3.0				1077, 2.0				257, 1.6			
SOC	N of Events	N of Patients	% of Patients	Rate/100 Pt-Yrs	N of Events	N of Patients	% of Patients	Rate/100 Pt-Yrs	N of Events	N of Patients	% of Patients	Rate/100 Pt-Yrs
**Total**	**3017**	**547**	**61.5%**	**105**	**1212**	**357**	**67.4%**	**113**	**212**	**93**	**59.2%**	**82.6**
Cardiac disorders	45	39	4.4%	1.56	20	16	3.0%	1.86	1	1	0.6%	0.39
Eye disorders	73	47	5.3%	2.53	25	15	2.8%	2.32	2	2	1.3%	0.78
Gastrointestinal disorders	193	112	12.6%	6.69	73	52	9.8%	6.78	12	7	4.5%	4.68
General disorders and administration site conditions	297	175	19.7%	10.3	169	148	27.9%	15.7	40	38	24.2%	15.6
Infections and infestations	689	275	30.9%	23.9	378	173	32.6%	35.1	62	36	22.9%	24.2
Injury, poisoning and procedural complications	178	108	12.1%	6.17	56	36	6.8%	5.2	17	11	7.0%	6.62
Investigations	7	58	6.5%	2.56	13	10	1.9%	1.21	5	4	2.5%	1.95
Musculoskeletal and connective tissue disorders	486	152	17.1%	16.8	130	84	15.8%	12.1	17	11	7.0%	6.62
Neoplasms benign, malignant and unspecified	54	46	5.2%	1.87	26	23	4.3%	2.41	1	1	0.6%	0.39
Nervous system disorders	166	107	12.0%	5.75	64	48	9.1%	5.94	8	6	3.8%	3.12
Psychiatric disorders	19	17	1.9%	0.66	10	10	1.9%	0.93	2	2	1.3%	0.78
Respiratory, thoracic and mediastinal disorders	226	131	14.7%	7.83	73	44	8.3%	6.78	15	10	6.4%	5.84
Skin and subcutaneous tissue disorders	260	160	18.0%	9.01	83	57	10.8%	7.71	14	11	7.0%	5.45
Surgical and medical procedures	42	38	4.3%	1.45	10	9	1.7%	0.93	1	1	0.6%	0.39
Vascular disorders	90	63	7.1%	3.12	14	14	2.6%	1.3	1	1	0.6%	0.39

**Table 4:** Serious adverse events occurring in ≥0.5% of patients per agent

	IFX (***N*** = 890)				GLM (***N*** = 530)				GLM-IV (***N*** = 157)			
Exposure (Total, Mean pt.yrs)	2714, 3.0				1077, 2.0				257, 1.6			
SOC	N of Events	N of Patients	% of Patients	Rate/100 Pt-Yrs	N of Events	N of Patients	% of Patients	Rate/100 Pt-Yrs	N of Events	N of Patients	% of Patients	Rate/100 Pt-Yrs
**Total**	**338**	**189**	**21.2%**	**11.7**	**121**	**82**	**15.5%**	**11.2**	**12**	**6**	**3.8%**	**4.68**
Cardiac disorders	20	18	2.0%	0.69	12	10	1.9%	1.11	0	0	0	0
Gastrointestinal disorders	12	10	1.1%	0.42	5	4	0.8%	0.46	2	1	0.6%	0.78
General disorders and administration site conditions	20	19	2.1%	0.69	7	7	1.3%	0.65	1	1	0.6%	0.39
Infections and infestations	77	58	6.5%	2.67	24	20	3.8%	2.23	3	2	1.3%	1.17
Injury, poisoning and procedural complications	30	21	2.4%	1.04	12	7	1.3%	1.11	1	1	0.6%	0.39
Metabolism and nutrition disorders	8	5	0.6%	0.28	1	1	0.2%	0.09	0	0	0	0
Musculoskeletal and connective tissue disorders	37	25	2.8%	1.28	12	10	1.9%	1.11	0	0	0	0
Neoplasms benign, malignant and unspecified	45	40	4.5%	1.56	16	14	2.6%	1.49	1	1	0.6%	0.39
Nervous system disorders	18	16	1.8%	0.62	10	9	1.7%	0.93	1	1	0.6%	0.39
Renal and urinary disorders	2	2	0.2%	0.07	4	3	0.6%	0.37	0	0	0	0
Respiratory, thoracic and mediastinal disorders	25	20	2.2%	0.87	3	3	0.6%	0.28	1	1	0.6%	0.39
Skin and subcutaneous tissue disorders	5	5	0.6%	0.17	2	2	0.4%	0.19	1	1	0.6%	0.39
Surgical and medical procedures	10	9	1.0%	0.35	1	1	0.2%	0.09	1	1	0.6%	0.39
Vascular disorders	9	8	0.9%	0.31	0	0	0	0	1	1	0.6%	0.39
Cardiac disorders	20	18	2.0%	0.69	12	10	1.9%	1.11	0	0	0	0

**Table 5:** Adverse events (preferred term; ≥2 patients with one agent)

	IFX (***N*** = 890)				GLM (***N*** = 530)				GLM-IV (***N*** = 157)			
	N of Events	N of Patients	% of Patients	Rate/100 Pt-Yrs	N of Events	N of Patients	% of Patients	Rate/100 Pt-Yrs	N of Events	N of Patients	% of Patients	Rate/100 Pt-Yrs
**Gastrointestinal disorders**												
Diarrhea	27	20	2.2%	0.94	12	12	2.3%	1.11	1	1	0.6%	0.39
Nausea	54	44	4.9%	1.87	13	12	2.3%	1.21	2	2	1.3%	0.78
Vomiting	23	20	2.2%	0.80	7	7	1.3%	0.65	1	1	0.6%	0.39
**General disorders and administration site conditions**												
Chest discomfort	26	22	2.5%	0.90	0	0	0	0	0	0	0	0
Chest pain	21	19	2.1%	0.73	2	2	0.4%	0.19	1	1	0.6%	0.39
Drug effect decreased	5	5	0.6%	0.17	22	22	4.2%	2.04	6	6	3.8%	2.34
Drug ineffective	19	19	2.1%	0.66	64	63	11.9%	5.94	23	23	14.6%	8.96
Fatigue	41	33	3.7%	1.42	5	5	0.9%	0.46	3	3	1.9%	1.17
Influenza-like illness	15	11	1.2%	0.52	16	15	2.8%	1.49	3	3	1.9%	1.17
Pain	23	20	2.2%	0.80	3	3	0.6%	0.28	0	0	0	0
Pyrexia	27	26	2.9%	0.94	4	4	0.8%	0.37	1	1	0.6%	0.39
Therapeutic response decreased	22	22	2.5%	0.76	23	23	4.3%	2.14	1	1	0.6%	0.39
**Infections and infestations**												
Bronchitis	51	41	4.6%	1.77	18	17	3.2%	1.67	5	5	3.2%	1.95
Ear infection	21	14	1.6%	0.73	11	10	1.9%	1.02	4	4	2.5%	1.56
Herpes Zoster	19	19	2.1%	0.66	14	13	2.5%	1.3	1	1	0.6%	0.39
Influenza	36	29	3.3%	1.25	13	10	2.1%	1.21	0	0	0	0
Pneumonia	47	41	4.6%	1.63	13	11	2.1%	1.21	2	2	1.3%	0.78
Sinusitis	53	31	3.5%	1.84	14	13	2.5%	1.3	8	6	3.8%	3.12
Upper respiratory tract infection	72	49	5.5%	2.49	57	45	8.5%	5.29	3	3	1.9%	1.17
Urinary tract infection	51	32	3.6%	1.77	32	23	4.3%	2.97	6	6	3.8%	2.34
**Injury, poisoning and procedural complications**	178	108	12.1%	6.17	56	36	6.8%	5.2	17	11	7.0%	6.62
Fall	24	21	2.4%	0.83	9	9	1.7%	0.84	9	4	2.5%	3.51
Infusion-related reaction	53	37	4.2%	1.84	0	0	0	0	0	0	0	0
**Musculoskeletal and connective tissue disorders**	486	152	17.1%	16.8	130	84	15.8%	12.1	17	11	7.0%	6.62
Arthralgia	150	60	6.7%	5.20	24	19	3.6%	2.23	5	5	3.2%	1.95
Back pain	30	26	2.9%	1.04	7	6	1.1%	0.65	0	0	0	0
Pain in extremity	65	30	3.4%	2.25	4	4	0.8%	0.37	2	2	1.3%	0.78
Osteoarthritis	26	18	2.0%	0.90	17	14	2.6%	1.58	0	0	0	0
Rheumatoid arthritis	57	37	4.2%	1.97	18	16	3.0%	1.67	3	3	1.9%	1.17
**Nervous system disorders**	166	107	12.0%	5.75	64	48	9.1%	5.94	8	6	3.8%	3.12
Dizziness	29	23	2.6%	1.00	5	5	0.9%	0.46	1	1	0.6%	0.39
Headache	61	44	4.9%	2.11	10	11	1.9%	1.02	1	1	0.6%	0.39
**Respiratory, thoracic and mediastinal disorders**	226	131	14.7%	7.83	73	44	8.3%	6.78	15	10	6.4%	5.84
Cough	40	28	3.1%	1.39	21	16	3.0%	1.95	3	3	1.9%	1.17
**Skin and subcutaneous tissue disorders**	260	160	18.0%	9.01	83	57	10.8%	7.71	14	11	7.0%	5.45
Pruritus	35	32	3.6%	1.21	2	2	0.4%	0.19	1	1	0.6%	0.39
Psoriasis	10	9	1.0%	0.35	16	11	2.1%	1.49	1	1	0.6%	0.39
Rash	39	32	3.6%	1.35	15	15	2.8%	1.39	2	1	0.6%	0.39
**Vascular disorders**	90	63	7.1%	3.12	14	14	2.6%	1.3	1	1	0.6%	0.39
Hypertension	27	22	2.5%	0.94	5	5	0.9%	0.46	0	0	0	0

**Table 6:** Adverse events of interest (preferred terms; malignancies in ≥2 patients, serious infections in ≥2 patients, Herpes Zoster, tuberculosis and opportunistic infections)

	IFX (***N*** = 890)				GLM (***N*** = 530)				GLM-IV (***N*** = 157)			
	N of Events	N of Patients	% of Patients	Rate/100 Pt-Yrs	N of Events	N of Patients	% of Patients	Rate/100 Pt-Yrs	N of Events	N of Patients	% of Patients	Rate/100 Pt-Yrs
**Malignancies**												
Acrochordon	2	2	0.2%	0.07	0	0	0	0	0	0	0	0
Basal cell carcinoma	2	1	0.1%	0.07	2	2	0.4%	0.19	0	0	0	0
Breast cancer	5	5	0.6%	0.17	2	2	0.4%	0.19	0	0	0	0
Leukemia	0	0	0	0	3	2	0.4%	0.28	0	0	0	0
Lung adenocarcinoma	1	1	0.1%	0.03	2	2	0.4%	0.19	0	0	0	0
Lymphoma	2	2	0.2%	0.07	0	0	0	0	0	0	0	0
Non-Hodgkin’s lymphoma	2	2	0.2%	0.07	0	0	0	0	0	0	0	0
Renal cell carcinoma	2	2	0.2%	0.07	0	0	0	0	0	0	0	0
Squamous cell carcinoma	4	4	0.4%	0.14	0	0	0	0	0	0	0	0
Uterine cancer	2	2	0.2%	0.07	1	1	0.2%	0.09	0	0	0	0
**Serious infections**												
Arthritis bacterial	4	3	0.3%	0.14	2	2	0.4%	0.19	0	0	0	0
Cellulitis	6	6	0.7%	0.21	1	1	0.2%	0.09	0	0	0	0
Pneumonia	23	19	2.1%	0.80	5	5	0.9%	0.46	1	1	0.6%	0.39
Pyelonephritis	1	1	0.1%	0.03	3	2	0.4%	0.28	0	0	0	0
Sepsis	3	3	0.1%	0.03	0	0	0	0	0	0	0	0
Urosepsis	2	2	0.2%	0.07	1	1	0.2%	0.09	0	0	0	0
**Herpes Zoster, tuberculosis and opportunistic infections**												
Herpes Zoster	19	19	2.1%	0.66	14	13	2.5%	1.30	1	1	0.6%	0.39
Tuberculosis (disseminated)	1	1	0.1%	0.03	0	0	0	0	0	0	0	0
Candidiasis	4	4	0.4%	0.14	0	0	0	0	0	0	0	0
Histoplasmosis	1	1	0.1%	0.03	0	0	0	0	0	0	0	0
Onychomycosis	2	2	0.2%	0.07	0	0	0	0	0	0	0	0

There were 18 deaths during the study among IFX-treated patients (0.66/100 pt.yrs). Cause of death included major adverse cardiovascular event (MACE; × 3), lung cancer (× 2), pulmonary fibrosis (× 2), pneumonia (× 2), respiratory failure, bronchitis, intestinal cancer, throat cancer, intestinal gangrene, disseminated TB, septic shock, procedural complications and unknown (one of each). Seven GLM-treated patients also died (0.64/100 pt.yrs). Cause of death were MACE (× 3), lung cancer (× 2), and unknown (× 2). One GLM-IV patient died from a MACE (0.25/100 pt.yrs).

## Discussion

Differences are found in patient characteristics between registries and randomized control studies [4], and the former are essential to determine the effectiveness and safety of new therapies in a broad, generalizable population. In the past decades, national and regional registries were established to evaluate anti-TNF agents in the treatment of RA [10]. However, most evaluated the earliest agents, such as IFX and etanercept, and only a few published registries included data on the newer anti-TNFs such as adalimumab [11, 12], certolizumab-pegol [13, 14] and GLM [15]. BioTRAC was one of the longest running RA registries and included data on both old (IFX) and new (GLM) anti-TNF agents.

When anti-TNFs were first approved for the treatment of RA, they were initially used in more refractory patients with longer established disease and higher disease activity. As time passed, they were used earlier, in more moderate activity patients. This can be seen if one compares the baseline characteristics of patients in the registration studies for IFX and GLM [16, 17]. Such a pattern, in which baseline disease activity decreased over time, had been reported in the interim analysis of the IFX-treated patients in BioTRAC [6]. Despite this, it was interesting to notice that baseline disease characteristics of the GLM-treated patients from 2010 to 2012 suggest that the first patients to be treated with GLM may have had more active disease than IFX-treated patients. This could be the result of an unconscious channeling bias towards using newer therapies in more severe patients, as the MDGA scores were identical between the two cohorts. Another possibility is that this was driven by the limited availability of the GLM auto-injector during that period, forcing the use of pre-filled syringes by most patients, along with uncertainties in market dynamics caused by the corporate takeover of Schering-Plough by Merck and the subsequent transition of the immunology portfolio to Janssen. Studies to evaluate the impact of disease duration, baseline disease activity and the adherence to treat-to-target guidelines on long-term function and outcomes are ongoing.

Despite difference in baseline disease activity, all three anti-TNFs showed efficacy with decreased disease activity and improved function. The route of administration does not appear to bring any specific efficacy benefit, as the data curves for GLM and GLM-IV patients are basically superimposable. Differences in the proportion of patients achieving target-specific outcomes such as LDA and remission were noted between IFX- and GLM−/GLM-IV-treated patients. Because we are reporting observed data, these differences could be driven by differences in baseline disease activity, the implementation of treat-to-target guidelines or the use of more stringent targets, such as remission rather than LDA, in later years when GLM and GLM-IV were more likely to be chosen as treatment. Also, the greater availability of additional treatment options could lead to a higher probability of switching therapies if such targets were not achieved. Therefore, caution should be exercised when interpreting the relative effectiveness of the three agents.

The incidence of AEs and SAEs was found to be similar between agents, although there were some notable differences. Patients treated with IFX had a greater incidence of chest discomfort, chest pain, fatigue, headaches, pain, pyrexia, pain in extremities and pruritus compared to GLM and GLM-IV patients, all of which could be due to acute and delayed infusion reactions [18]. Conversely, GLM and GLM-IV patients had a greater incidence of “lack of response” or “loss of response” AEs compared to IFX-treated patients, although this was likely driven by changes in the “End Of Participation” questionnaire and the addition of lack/loss of response as an AE of special interest in a protocol amendment after 2014 (see below).

The incidence of serious infections was 1.2–2.7 events/100 pt.yrs., slightly lower than the incidence of 4–4.4 events/100 pt.yrs. reported in other registries [10, 11, 19]. However, since anti-TNF therapy in RA patients was associated with an increased risk of serious infections, especially in the first 6 months of treatment [20, 21], registries with very long duration of follow-up would have a tendency to report a lower incidence rate. The low incidence of serious infection could also be explained by the low level of disease activity achieved and maintained over time. Indeed, the CORRONA registry assessed the relationship between DAS28 and infection in RA patients and found that high disease activity was associated with an increased risk of infection [22]. Analyses from the BSRBR and Italian LORHEN registries showed similar results [20, 23]. However, other European registries suggested that higher disease activity as measured by DAS28 was not directly associated with an increased incidence of serious infections [24]. Post Hoc analyses could be done in order to determine if serious infections are linked to control of disease activity, age, the use of concomitant MTX, glucocorticoids or survival bias from dropout of patients who developed an infection and subsequently stopped their anti-TNF.

The limitations of this registry are the absence of a non-biologic DMARD control group, the inclusion of predominantly bio-naïve patients and the inherent biases that are common within non-interventional, observational studies. Other limitations are related to non-inclusion of specific data sets that were not “standard of care” among community clinics in the mid-2000’s as this would have led to many missing data points. Examples of these includes radiographic imaging, the complete 66/68 joint count and baseline co-morbidities (although smoking habits were recorded since 2009). Also, the long duration of the registry could have had an impact on data quality over time due to protocol amendments, changes in standard operating procedures from the three sponsors and improvements in adverse event reporting from refining processes and increasing site experience. An example of the above was site training implemented in 2014 following the first interim analysis of the IFX cohort [6] to limit the inappropriate use of the “Other reason; provide details” box within the “End of participation” form when patients were losing response. This led to an increase in the incidence of lack/loss of response AE reporting in later years which had a larger proportion of GLM- and GLM-IV-patients.

Also, despite its respectable size, BioTRAC had limited ability to detect rare AEs unlike large national registries, such as the UK’s BSRBR, Sweden’s ARTIS, Germany’s RABBIT, Denmark’s DANBIO, Spain’s BIOBADASER and the US’s CORRONA [10]. Indeed, most Canadian multi-center registries, such as BioTRAC, CATCH [25], OBRI [26] and RHUMADATA [27], are smaller in scope but still provide significant insights on the treatment of RA at a regional level. CATCH, OBRI and RHUMADATA have the advantage over BioTRAC of being disease registries enrolling RA patients taking any therapy (biologic and non-biologic DMARDs). CATCH is an early RA disease registry enrolling newly diagnosed RA patients while OBRI and RHUMADATA enrolls RA patients from academic and community centers but are restricted to the provinces of Ontario and Quebec, respectively [26, 27]. Despite those differences in design, it has been possible to increase power and answer specific scientific questions by combining patient data from multiple registries [28].

One key strength of BioTRAC is that it included an extensive evaluation of clinical disease parameters, most of which were not collected elsewhere, especially in the early years [10]. Due to its long-term duration, BioTRAC offered a unique opportunity to evaluate the real-world effectiveness and safety of three anti-TNF agents in a community Canadian setting, while assessing regional variations due to differences in patient profiles, practice patterns and local reimbursement policies impacting access to care over 16 years. Although there has been extensive real-world evidence generated on the early anti-TNF agents such as IFX or etanercept, very little efficacy data has been published with other anti-TNF agents such as GLM, and most of those only presented persistence data [15, 29–31]. One exception, however, is the GO NICE prospective non-interventional trial in Germany for inflammatory arthritis patients treated with GLM [15, 32]. This 2-year trial also found significant clinical effectiveness among RA patients [15], as well as improvements in patient-reported health status, physical function, and fatigue levels [32].

## Conclusion

In conclusion, this real-world study identified differences in baseline characteristics between Canadian RA patients treated with an anti-TNF over time and between agents. The study also revealed potential biases when selecting a given therapy which may impact the proportion of patients achieving a target-specific outcome. Finally, treatment with IFX, GLM and GLM-IV significantly reduced disease activity and improved functionality in a similar fashion and all agents were safe and well- tolerated.

## Supplementary information

**Additional file 1: Supplemental Table 1.** Discontinuations and reasons for discontinuations with IFX between 2010 and 2014 and with GLM. **Supplemental Figure 1.** Time to Discontinuation Due to Lack/Loss of Efficacy or Disease Progression between 2010 and 2014 with IFX vs. GLM.

## Data Availability

Janssen has an agreement with the Yale Open Data Access (YODA) Project to serve as the independent review panel for evaluation of requests for CSRs and participant level data from investigators and physicians for scientific research that will advance medical knowledge and public health. For more information on this process or to make a request, please go to https://yoda.yale.edu/.

## Abbreviations

AE: Adverse event
AM: Morning
bDMARD: Biologic DMARD
BioTRAC: Biologic Treatment Registry Across Canada
CRP: C-Reactive protein
DMARD: Disease-modifying antirheumatic drug
ESR: Erythrocyte sedimentation rate
GLM: Golimumab
GLM-IV: Golimumab intravenous
IFX: Infliximab
MTX: Methotrexate
HAQ-DI: Health Assessment Questionnaire Disease Index
MACE: Major adverse cardiovascular event
MDGA: Physician global assessment of disease activity
PtGA: Patient global assessment of disease activity
RA: Rheumatoid arthritis
RCT: Randomized-controlled trial
SAE: Serious adverse event
SD: Standard deviation
SJC28: Swollen joint count based on 28 joints
TJC28: Tender joint count based on 28 joints
