# Supplementary material for: Long-term effectiveness and safety of infliximab, golimumab and golimumab-IV in rheumatoid arthritis patients from a Canadian prospective observational registry
Source: BMC Rheumatol. 2020 Sep 19;4:46. doi: 10.1186/s41927-020-00145-4 (PMC7501619; doi:10.1186/s41927-020-00145-4)
Supplement: Supplementary file 1 — Additional file 1: Supplemental Table 1. Discontinuations and reasons for discontinuations with IFX between 2010 and 2014 and with GLM. Supplemental Figure 1. Time to Discontinuation Due to Lack/Loss of Efficacy or Disease Progression between 2010 and 2014 with IFX vs. GLM. [file 41927_2020_145_MOESM1_ESM.docx]

**Supplemental Table 1: Discontinuations and reasons for discontinuations with IFX between 2010 and 2014 and with GLM**

|  | **IFX**  **2010-2014** | **GLM**  **2010-2014** | **GLM**  **2015-2017** |
| --- | --- | --- | --- |
| **Total discontinuations (n/N, %)** | 98/149, 65.8% | 178/301, 59.1% | 102/229, 44.5% |
|  |  |  |  |
| **Reason for discontinuation (n, %*)** |  |  |  |
| Patient withdrew consent | 4, 4.1% | 18, 10.1% | 7, 6.9% |
| Adverse event | 31, 31.6% | 22, 12.4% | 11, 10.8% |
| Lost to follow-up | 6, 6.1% | 20, 11.2% | 8, 7.8% |
| Financial reasons | 3, 3.1% | 4, 2.2% | 0, 0.0% |
| Complete response | 0, 0.0% | 4, 2.2% | 0, 0.0% |
| Disease progression | 1, 1.0% | 7, 3.9% | 7, 6.9% |
| Lack of response | 13, 13.3% | 32, 18.0% | 35, 34.3% |
| Loss of response | 10, 10.2% | 31, 17.4% | 15, 14.7% |
| Unusual lack of efficacy | 0, 0.0% | 0, 0.0% | 1, 1.0% |
| Geographic issues | 1, 1.0% | 2, 1.1% | 1, 1.0% |
| Patient switched to another therapy | 10, 10.2% | 12, 6.7% | 3, 2.9% |
| Did not meet entry criteria | 0, 0.0% | 0, 0.0% | 0, 0.0% |
| Other | 19, 19.4% | 26, 14.6% | 14, 13.7% |
| Missing | 0, 0.0% | 0, 0.0% | 0, 0.0% |

*Proportions based on number of discontinued patients; GLM-IV patients are not shown due to the fact that GLM-IV was only included in the registry in 2014

**Supplemental Figure 1: Time to Discontinuation Due to Lack/Loss of Efficacy or Disease Progression between 2010 and 2014 with IFX vs. GLM**


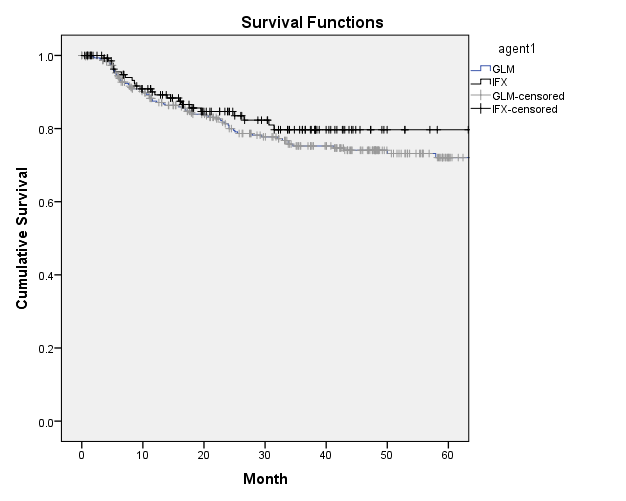


*p=0.292
